# Innovation for sustainability: how actors are myopically caught in processes of co-evolution

**DOI:** 10.1098/rstb.2022.0270

**Published:** 2024-01-01

**Authors:** René Kemp, Harro van Lente

**Affiliations:** ^1^ UNU-MERIT, Maastricht University, Maastricht, Limburg 6211 AX, The Netherlands; ^2^ MSI, Maastricht University, Maastricht, Limburg 6211 AX, The Netherlands; ^3^ FASOS, Maastricht University, Maastricht, Limburg 6211 AX, The Netherlands

**Keywords:** sustainability, co-evolution, governance, modulation, Anthropocene

## Abstract

In this paper, we argue that the development, uptake and adoption of innovations resembles an evolutionary process of variation, selection and retention (within broader processes of co-evolution) in which actors are myopically caught. We do so in four steps. First, we review in what ways socio-technical evolution resembles biological evolution. Second, we argue that in socio-technical evolution so-called ‘configurations that work’ can be viewed as evolutionary units, which are subject to selection pressures, variation and human-made couplings between variation and selection. This explains why innovation is often cumulative, based on variation and recombination. Third, we discuss how producers, consumers, governments and scientists are myopically caught in processes of co-evolution. While humans are capable of imagining the need for system change and details of desired systems, they are less capable of accepting the concomitant higher costs and inconveniences and adopt new interpretive schemes. Fourth, in a pluralist world, steering is done by all kind of actors, including those who actively resist transformative change. Because of this, steering by government and coalitions of change can achieve little more than a modulation of ongoing dynamics, despite disturbing evidence of a run-away climate, mass extinction, pervasive ecological degradation and steady depletion of resources. A new consciousness of the Anthropocene can evoke fundamental changes in science and the economy if—and only if—they are sufficiently carried by institutional changes and new practices.

This article is part of the theme issue ‘Evolution and sustainability: gathering the strands for an Anthropocene synthesis’.

## 1. Introduction: innovation as evolution

Modern societies rely for their well-being and sustenance on natural resources and ecosystems, but natural resource pools and environments have been degraded and the climate system is disturbed by greenhouse gas emissions [1]. The need for change is clear and accepted by decision makers (Paris Agreement of 2015, the European Union (EU) Green Deal), but humans have great difficulties in achieving projected futures of, say, a decarbonized, circular economy and regenerative agricultural system [2]. This difficulty is not so much a problem of knowledge and skills, but much more a problem of altering co-evolution and governance patterns [3]. The aim of this paper is to disentangle this problem.

This paper approaches the issue of evolution and sustainability in the Anthropocene from the angle of evolutionary innovation studies and transition studies [4,5] that view innovation *as an evolutionary process* and are based on a *co-evolutionary* perspective on socio-technical transitions. According to this literature, the creation and uptake of innovations (new products and production technologies) resembles a process of variation, selection and retention. For a long time, this resemblance has caught the attention of historians of technology [6] and innovation scholars [7], as well as theorists on the evolution of human culture [8]. New products and processes, then, are seen as variations on earlier offerings by firms that struggle for survival in selection environments like the market and regulatory regimes. A summary of the literature on technology as an evolutionary process is offered by Grodal *et al.* [9], who distinguish four perspectives on evolution of innovation: technology-realist, economic realist, cognitive interpretivist and social constructivist.

In this literature, more attention is given to the *similarities* than to the differences between socio-technical and biological evolution. Yet, differences from biological evolution are apparent, too, in particular with the Modern Synthesis, which attributes a great role to natural selection as the major directing influence on evolution, random genetic variation, gradualism and macro-evolution as the outcome of micro-evolution [10].^1^ The main differences are that variation is not random as clearly some anticipation of selection criteria occurs in variation generation: selection pressures can be changed via the wilful enactment of selection criteria via regulation and calls for change; retention is not firmly secured in DNA, but in inherited mind sets, material technologies, skills, habits, identities and roles. These key differences imply that the evolutionary processes of innovation are complex and subject to path dependence and that attempts to ‘manage’ these are often illusory [13], a topic discussed in §4. Whilst the differences from the Modern Synthesis are notable, the differences from current understandings of biological evolution are less striking. Drawing on a wide range of research (for example, [14,15]), Laland *et al*. [10] advanced an ‘extended evolutionary synthesis’ to accommodate novel biological insights regarding developmental bias and plasticity, inclusive inheritance and niche construction. Their framework of biological evolution involves ‘constructive development’ (the ability of an organism to shape its own developmental trajectory) and ‘reciprocal causation’ (organisms as outcomes of evolution are not solely products but also causes of evolution). These new insights of biology often have their parallels in innovation, such as the importance of symbiosis. Symbiosis helps to make bigger steps than what both organisms and organizations are individually capable of, and helps to explain the emergence of new systems, as the cumulative result of coordinated action (over an extended time) and helpful external developments.

We start this paper by discussing co-evolutionary models of technological and economic change and discuss the literature on the evolution of needs (how these are shaped by business and more recently by social movements) and the role of various forms of assessment and imagination in shaping evolutionary dynamics. We outline a configurational perspective on technology, as ‘configurations that work’ whose creation and evolution are shaped by social, economic and political forces with whom they interact. In §3, we discuss how the evolution of innovation relates to sustainability and put forward a dynamic sustainability view, which contends that sustainability is not a state but reflects the consensus on relevant problems. In §4, we discuss various forms of steering. We conclude that traditional steering attempts by government could benefit from the idea of modulated evolution via co-evolutionary governance. Section 5 offers a short summary of our main arguments regarding the evolutionary nature of innovation and the possibilities and limitations of steering approaches for sustainability.

## 2. Co-evolutionary approaches to innovation

The evolutionary nature of innovation and development is well-studied and conceptualized in evolutionary economics and sustainability transition studies that are engaged with processes behind path dependence and path creation [4,16,17]. A study of Kemp & Turkeli [18], on the use of ecological concepts in the economics literature, revealed that the most widely used ecological concepts are: evolutionary (19670), eco-system (5033), helix (1099) and co-evolution (888), while the terms 'variation', 'selection' and 'retention' were used 124, 317 and 39 times. Also, in complexity theory [19], an explicit evolutionary approach is adopted based on the so-called fitness landscape and the ‘adjacent: possible’, ‘the set of possibilities available to individuals, communities, institutions, organisms, productive processes, etc. at a given point in time during their evolution’ [20, p. 9]. The adjacent possible depends on imagination and (individual and collective) assessments of possibilities. The ideas of fitness and path dependence [21,22] help to explain that innovations that are an improvement of what exists or a recombination of configurations (*neuer Kombinationen*) are relatively easily achievable, whereas transformative innovations (involving fundamental change in social relations and new system architectures) are not.

In the field of sustainability transitions [23–26], the dynamics of adaptation and transformation in sectors are studied.^2^ The role of imaginaries and projective agency is studied in science, technology and society studies (STS) [27] and the co-evolution of society and economy in studies on development and institutional change. In those literatures, the following models of co-evolution are distinguished: coupled dynamics of sociosystems and ecosystems [28], supply and demand [29], technology, industry structure and institutions [30,31], technology and society and governance [32,33], actor/institution configurations and power/knowledge configurations [34], policy (mixes) and socio-technical systems [35] and science, technology and society [36].

In evolutionary (innovation) economics and sustainability transition studies the notion of co-evolution is key, but remarkably weakly defined. We propose to define co-evolution as a condition in which two or more processes with evolutionary elements of variation, selection and retention influence each other through *selection pressures* and *resourcing*. An example of selection pressure is climate policies coercing business to reduce greenhouse gas emissions; an example of resourcing is the provision of subsidies and informational support. The evolution of business is thus directly connected to the evolution of government policies. As all societal sectors (including the government sector, finance and science) show interdependency, within those sectors and between sectors, any sector co-evolves with other sectors. Also the wider institutional order (trade regimes, democracies, and rights and obligations for citizens and business) is subject to co-evolutionary dynamics and meta-governance (the *governance of governance* [37]).

The insight that variation and selection are coupled in various ways is nowadays widely shared among innovation scholars. The coupling consists of anticipation of regulation, innovation possibilities and competition, and attempts to influence selection pressures by lobbyists who oppose regulation and by innovation advocates who seek support for their innovations and visions for society. Test labs and technology platforms are vehicles for coupling variation and selection. According to the literature, variation, selection and retention can be coupled in the following ways:

— When creating novelty, firms anticipate regulation and other changes in the selection environment.
— Governmental innovation programmes offer resources to foster advances in an innovation field (thus softening selection pressures for certain variations).
— New institutional logics [38] and selection criteria (evaluative values) get internalized by subjects, who obtain different dispositions and schemes of evaluation [39].^3^
— Imaginaries alter the realm of the desirable and conceivable [40], giving rise to promises-requirements cycles [41].
— Policy brokers, platforms and intermediaries act as interconnectors between variation and selection, especially for symbiotic and architectural innovations [42].
— The selection environment is human-made in important ways: via rule systems and markets and trading regimes that have been established.

Owing to these couplings, the interaction of actors in various sectors and governance systems gives rise to evolutionary patterns, and results in trajectories that, in turn, exercise selective pressures on radical novelties that seek to break away from those trajectories. However, in some places (known as ‘niches’) selection pressure and local resources are congenial to the development and spread of radical novelties [43,44]. Niches may be actively created. Examples are sites of experimentation where actors test and improve new configurations and engage in collective action.

### (a) Configurations that work

In our own work [32,45], we outlined a configurational perspective on technology to accommodate these insights. We characterized technologies as ‘configurations that work’, whose creation and development is shaped by social, economic and political forces with which they interact. Innovation is part of journeys [46] and entanglements [47] that are played out in various ways: via novelty creation, adaptation of systems and regimes, and transformative change. Identifiable ‘configurations that work’ could be viewed as evolutionary units with an underlying structure: a genotype as a set of instructions and practical knowledge, and a manifest entity (phenotype). This structure involves organizational socio-technical activities and firm-specific beliefs and injunctions of ‘how we do things here’ and shared expectations about possibilities and beliefs about what customers value. They are reflected in ‘configurations that work’, which are the basis for improvement strategies. Such combinations of technology, practices, frames of evaluation and associated capabilities and roles can be viewed as an evolutionary unit that is evolving over time (subject to variation/elaboration, wider diffusion or replacement by other configurations).

We consider evolutionary models of innovation that view information (memes) and habits or routines [29] as the unit of evolution as too limited. Compared with biological units, variation, selection and retention of man-made configurations are much more coupled and capable of recombination (technically and organizationally). The configurations are part of regimes or niches and shaped by background dynamics with which they interact. In general we observe that such interdependences are often poorly conceptualized because science is fragmented. This is especially true for disciplinary science, where economists and engineers tend to look at resource flows, technologies and markets only, while political scientists look at political struggles over policies, and socio-biologists and neuroeconomists at the role of instincts and neural circuitry.^4^

### (b) The Anthropocene

The evidence of a growing impact of humans on the environment (with ecosystems being damaged and overexploited by human activities) led to the notion of 'the Anthropocene' to describe the present period in Earth's history, when human activity has a significant impact on the planet's climate and ecosystems.^5^ Anthropocene refers to a condition (of humans as geological agents), to a new object of knowledge and to an order of governance [50].^6^

Scientists interested in Earth system governance research [52] hope that the Anthropocene concept will help the research community ‘to move toward new conceptual syntheses and integrative action-oriented approaches’ for dealing with the challenges of global change and sustainability [53, p. 318]. This can be done through attention to the ‘emergent properties of socio-ecological systems’, ‘urbanization and resource nexus’ and ‘systemic risks and tipping points’ [53, pp. 323–324]. Attention to complex human–environment relations and actionable models and approaches for transforming those relations into better ones are the hallmark of sustainability science [54] and socio-ecological transformation research [55,56]. Yet, such integrative science is up against serious barriers of funding and conservatism deeply built into science and education based on separate disciplines. As shown and discussed by MacLeod [57], large conceptual and methodological divides and conflicts over epistemic values work against collaboration between biologists and economists. The disciplinary organization of science, education and research acts as a strong barrier to interdisciplinary research.

## 3. Innovation for sustainability: a co-evolutionary perspective

An important empirical finding from innovation studies is that socio-technical regimes of production and consumption in energy, agrofood and mobility in modern societies are based on fossil fuels, and involve global chains and powerful actors who are resistant to fundamental change. This is especially true for car-based mobility and chemical industry products. A key reason is that certain products and processes achieve strong competitive advantages that create barriers to innovation for systems that have not achieved these [21,58].

Another key finding is that transformative change usually comes from outsiders (niche players) who have less of a vested interest in the status quo. Regime actors depend on a continuation of the existing earning model and face pressures from capital owners. They may even form a cartel of resistance.^7^ While they may be perfectly capable of looking far into the future, their decision making is myopic: focused on short-term gains. Messages about climate change and the Anthropocene compete and interact with other considerations.

A weakly developed aspect in the study of socio-technical change and the field of economics is the evolution of needs. This is particularly important when thinking about changes towards more sustainability. While the development of novelty has been studied extensively, the co-evolution of needs with new technologies has received much less attention. We lack a theory of changing aspirations, as needs are not pre-given but show important changes over time. The urge to take a bath, for instance, has dramatically changed in the last decades, to the current standard of having a daily shower [62]. Or how Hodgson [63, p. 117] has phrased it: ‘People do not develop new preferences, wants or purposes simply because ‘values’ or ‘social forces’ control them. What does happen is that the framing, shifting and constraining capacities of social institutions give rise to new perceptions and dispositions within individuals. Upon new habits of thought and behaviour, new preferences and intentions emerge’. The rise of mobility and the widespread use of the internet are acute examples of the establishment of new needs [64,65].

The notion of needs is notoriously elusive, as it points to physiological conditions, cultural wants, economic preferences and existential desires. Jolibert *et al.* [66], for instance, distinguish between needs, which are seen as fundamental and the same in every culture, and the various ways to satisfy them (satisfiers), which will vary across cultures. The philosopher René Girard coined the idea of ‘mimetic desire’ to elaborate how desires are always copied from someone else: we always need a model to decide what we desire [67]. Girard's insights resonate with cultural historians who point to the spread of consumption behaviours, such as Veblen and his *Theory of the leisure class* and the sociological analysis by Bourdieu of how consumption is related to status [68]. In a similar vein, Tilly [69, p. 55] speaks of an embourgeoisement of workers, who ‘in material possessions, leisure and personal style’ emulated those of the bourgeois. Pleasure seeking lifestyles, status goods and political consumption constitute examples of new types of demand, with advertising catering actively to the first two types of consumption. Changes in needs are not acts of will, but the outcome of social processes involving comparison with others.^8^ It is important to gain a better understanding of how needs relate to sustainable options as it is clearly not sufficient to assume that new technologies cater for pre-given needs.

The lock-in to particular products, technologies and ways in life can be countered via four strategies: (i) the use of end-of-pipe technologies (filters, catalytic converters and the capture and storage of CO_2_), (ii) the use of corporate, social responsibility (sustainability) systems (aimed at reducing the impact of existing products for instance via re-use, recycling and resource efficiency), (iii) sustainability transitions (replacement of products and associated technologies by those that are less environmentally harmful, such as electric cars and renewable energy technologies) and (iv) alternative lifestyles (downshifting with product sharing) and the production of modern energy by prosumers (either as individuals or within renewable energy cooperatives).

Mechanisms behind transformative innovation and sustainability transitions have been studied by innovation and transition researchers. The dynamics display evolutionary features of variation, selection and retention and cross-over effects. Hybrid forms and niche accumulation are common features in sustainability transitions [71], as shown by the example of electric mobility.^9^ The agency of actors is enacted in processes of co-evolution. When researchers, firms and governmental actors become aware of interdependencies and *act* upon understandings of complex interaction effects (for instance by engaging in system-building activities for transformative configurations such as battery electric cars), the co-evolution becomes less myopic and inward-looking [3]. Changing beliefs and meanings of what is normal, desirable, permissive and sustainable are co-constitutive elements of processes of co-evolution. The meaning of sustainability has been broadened to include 17 Sustainable Development Goals many of which have to do with reducing human depreciation and emancipation.

## 4. Sustainability and co-evolutionary steering

Sustainability problems are not something new and neither are responses to them. As a rule, such responses are riddled with paradoxes and dilemmas. An example is the responses to wood shortages in the country we live in (The Netherlands), which range from using toxic creosote oil to preserve wood, to shipping wood from other parts of the world and using fossil fuels as an energy source for heating.

Innovation as a response to sustainability questions is studied by sustainability transition scholars, who focus on the dynamic interplay between actors, technologies and institutions, which includes both formal institutions and institutional logics (frames of evaluation and organizational ways of doing). In their studies they tend to focus on regime actors and niche players and less on institutional logics. The rise of new concepts in society and the economy—such as resilience, circular economy and net zero forms of production—is an example of institutional change, but the short-term influence of concepts is small because of the dominance of existing institutional logics, the power of incumbents, and the cost advantage of ecologically harmful ways of producing.

On the issue of steering, experts disagree. Market-based instruments are strongly favoured by economists, as these are believed to achieve environmental benefits at the lowest cost and because they are supposed to offer a greater spur to innovation than regulation. Innovation scholars, in contrast, attest important roles to (i) creating positive conditions for innovation (a facilitating eco-system), (ii) collaboration between business, academia and government (the so-called 'triple helix') and intermediary organizations, and (iii) mission innovation policies [74,75].

Blueprints for a different economy and different sectors are seldom advocated as a solution strategy; even the book *Blueprint for a green economy* [76] is very open to the ways in which this is to be achieved. This hesitation refers to experiences with forced transformations that are very negative. In the book *Seeing like a state* [77], James Scott offers examples of state-initiated social engineering^10^ that failed because of a combination of the following four factors: (i) the administrative ordering of nature and society, (ii) a high-modernist ideology, (iii) an authoritarian state that is willing and able to use the full weight of its coercive power to bring these high-modernist designs into being, and (iv) a prostrate civil society that lacks the capacity to resist these plans (https://www.nateliason.com/notes/seeing-like-a-state-james-c-scott). The negative experiences with forced change have led some scholars to propose disjointed incrementalism [78], but there is also a middle-ground, in the form of *perspectivischer Inkrementalismus* or ‘radical incrementalism’ [79], a step-wise process into positive transformative directions.

A burgeoning literature exists on evolutionary steering and co-evolutionary governance. Van Assche *et al*. [80], for instance, developed an evolutionary theory of governance, which understands governance as radically evolutionary, including features such as path-dependency, goal-dependency with complex interaction effects, and an important role for the adjacent possible. For achieving sustainability transitions, various models of evolutionary steering have been proposed, which are aimed at *altering the dynamics of variation and selection* [81]. The modulation of dynamics can be done by nurturing variation and fostering coordination and adaptation of the selection environment through innovation policies and environmental policies and the creation of nexuses (design laboratories, platforms). Examples of evolutionary steering of innovation processes are strategic niche management [44] and time-strategic policies [82]. A more comprehensive approach for changing the dynamics of variation and selection is by setting long-term goals and co-managing portfolios of options in a forward-looking and adaptive way. In such a long-term change approach based on flexibility and adaptation, the role for governments is to mobilize actor networks and support research and innovation activities in promising paths. The two best-known approaches for this are transition management [83–85] and mission innovation policy [75]. In both approaches, a portfolio of options is being explored, tested and perfected and the use of those is governed by markets and public decision making. As a policy approach, the evolutionary approach has great advantage as it explicitly seeks to make use of opportunities and ongoing dynamics, which are *modulated* in the direction of desirable transformations, through institutional work by dedicated actors (leading to supportive policies and strategies to deal with barriers). It accepts that steering is done by different actors and does not entertain illusions of top-down steering and control (known as cockpit-ism) [86]. Illusions of steering are important for *doing* something and are thus productive (performative) in this regard [13].

A fundamental problem for steering societal processes is that those who steer are part of systems and processes they wish to steer. This will lead them to focus on aspects that matter to them, while neglecting the aspects that matter to others. Sovacool [87] studied the literature on the victims of energy transitions and identified four processes responsible for negative outcomes: enclosure (capture of land or resources), exclusion (unfair planning), encroachment (destruction of the environment) or entrenchment (worsening of inequality or vulnerability). In India, for example, tree plantations for fuelwood or the cultivation of *Jatropha* for biofuel gave rise to all four processes: the dispossession of communities living near forests (enclosure), a government process of target setting that marginalized stakeholders (exclusion), diversion of water for irrigation (encroachment) and unfair patterns of property rights, land tenure and power (entrenchment). Transitions towards sustainability thus raise questions of fairness and equality.

In the global North, demands for a ‘just transition’ intend to soften the pain of a transition by addressing the demands from those affected, as well as their interests and well-being. Here, the science on transition policy also is offering cues. Rosenbloom *et al*. [26], for instance, harnessed insights on path dependence, policy feedback and transition pathways for energy transition policy. Subsequently, they present four proposals: (i) embed the low-carbon transition in a broader transformative agenda, (ii) build societal legitimacy for climate policy, (iii) encourage the growth of constituencies with a material interest in climate-friendly transformations and (iv) create a supportive eco-system of institutions. Their proposals helps to develop attractive ‘configurations that work’ as part of a (reflexive) co-evolutionary steering approach [3,88].

In business studies, scholars have put forward tools and frameworks to increase the chance of achieving successful innovation, through the use of experimentation, collaboration and advice to think ‘out of the box’. There is also a literature of intervention points for leveraging systemic change [89,90] drawing on [91]. Leverage points are ‘places within a complex system […] where a small shift in one thing can produce big changes in everything’ [91, p. 1]. Our co-evolutionary model is less optimistic about leveraging transformative change, but agrees that dynamics can be modulated.

The Sustainable Development Goals are an attempt to insert societal wants into decision making but transformative changes in the systems of production and consumption are needed to achieve those. This will require changes in practices, arrangements and new ways of thinking and evaluation that cannot be enforced. Changes in social practices are connected with regime changes, the creation of which can take decades and owes a lot to (political and socio-economic) landscape developments [92]. One could argue that civil society organizations and, to a smaller degree, scientists are less caught into socio-technical regimes than producers, consumers and policy makers, but even they are not masters of their thoughts, theories and methods. We are all perpetrators *and* victims of the very dynamics we seek to understand and transform. Actors are myopically caught in processes of co-evolution, not because they are shortsighted and stupid but because ‘unsustainability is the accumulated result of the improvement processes of many, intertwined, smart actors combined’ [93, p. 896]. Coupled dynamics based on imaginaries and supportive policies may result in new patterns of interaction across the world, with tentative rules and structures that institutionalize and guide subsequent actions and interactions [3].

## 5. To conclude

Socio-technical change and socio-ecological change have been successfully studied from an evolutionary perspective. Socio-technical evolution (the topic of this paper) resembles natural evolution in having similar building blocks but there are also important differences: (i) variation is informed by projected futures (a decarbonized economy, a more circular economy and the omnipresent use of artificial intelligence), which means that mutation is not random but channelled, (ii) selection pressures on products are to a large degree human-made (in the form of shareholder demands for profits, regulations, values of permissiveness and imaginaries that guide innovation decisions), (iii) the coupling between variation and selection is institutionalized, for instance via test labs, collaborative projects, the agency of intermediaries and innovation policies.

In this paper we put forward a co-evolutionary perspective which helps to understand myopia in socio-technical regimes and innovation-based possibilities to escape it. A co-evolutionary steering approach relies on: (i) making use of the dynamics and momentum of ongoing processes of change; (ii) inserting greater reflexivity in processes of problem solving and (iii) making sure that problem-solving approaches in one area do not aggravate problems in another area. Three ways to do this are joined-up thinking in policy, integrative forms of science and the use of system thinking in stakeholder processes. These help to create space for transformative action, based on the adjacent possible and imaginaries of progress.

According to biologists, humans ‘are torn, on the one hand, between what reason and moral judgment say we should do and what pure emotion and baser instincts compel us to do, particularly in stressful circumstances’ [94]. Such insights suggest that we are not rational observers of our behaviour and that we are not at the steering wheel of necessary changes but myopically caught in certain ways of doing and thinking, in habits and in socio-technical regimes that have achieved developmental advantages. These insights call for modesty, but also for courage to continue trying to respond. A new consciousness of the Anthropocene may evoke fundamental changes in science and the economy, but only when they are sufficiently carried by institutional changes and new practices.

## Data Availability

This article has no additional data.

## Endnotes

1 The Modern Synthesis is based on ‘the integration of Darwinian natural selection, population-level thinking and Mendelian inheritance’ according to Laland *et al.* [10, p. 1], referring to Provine [11] and Mayr [12] as landmark contributions.

2 The primary frameworks for analysis of niche innovations and regime changes are: strategic niche management, technology innovation systems, the multilevel perspective and social practices.

3 Examples of new criteria are: circularity as a design criterion for products, resilience, net-positive, shared value creation, just transition.

4 A discussion of sociobiology, evolutionary psychology, dual inheritance (gene–culture co-evolution) and mimetic thinking is offered by van den Bergh [48] and a discussion of techno-economic, socio-technical and political studies of the energy transition is provided by Cherp *et al.* [49].

5 See https://education.nationalgeographic.org/resource/anthropocene/.

6 It is an example of what sociologist Giddens [51] called a double hermeneutic: a concept from science becoming part of the vocabulary of others actors.

7 Regime resistance is played out in the following ways: by contesting evidence about problems, drawing attention to negative economic consequence and perceived injustices and unfairness, and by demands for self-regulation and reliance on the results of innovation (via general appeals to innovation or appeals to specific measures). Science does not offer a way out. As noted by Sarewitz [59, p. 385]: ‘science supplies contesting parties with their own bodies of relevant, legitimated facts about nature, chosen in part because they help make sense of, and are made sensible by, particular interests and normative frameworks’. Through privileged positions in systems of governance, regime actors are able to resist and undermine transformative change [60,61].

8 A discussion of the emergence of socially responsible consumption and its obstacles is offered by Schlaile *et al.* [70].

9 Electric mobility was pushed by the Californian Air Resources Board in the 1995–2005 periods, as a solution to the problem of smog, through mandatory car sales for zero emission vehicles and low emission vehicles for 1998–2003. The Board clashed with the car industry, who had to sell those cars at a loss, but the Californian policies and the unanticipated success of the Toyota Prius Car (a hybrid electric car) changed perceptions of car manufacturers, consumers and policy makers. Electric mobility became a strategic issue for the car industry and environmental and industrial policy makers. For China, the electric car offered an opportunity to obtain a foothold in the car market dominated by foreign companies. Advances in battery technology (which built on the battery market for laptops) helped to increase the range as an important consumer evaluation aspect. Prices of batteries dropped considerably during the 2011–2020 period thanks to technical advances, scale economies and competition (https://www.iea.org/data-and-statistics/charts/average-pack-price-of-lithium-ion-batteries-and-share-of-cathode-material-cost-2011-2021). The first electric cars were used by environmentally minded people; the next niche was that of luxury cars. Supply offerings have expanded considerably. Nowadays practically all car manufacturers are offering full battery electric cars, next to hybrid electric cars. In the European Union, from 2035 all cars and vans should be zero emission vehicles, offering further impetus to electric cars. The momentum of innovation has shifted from internal combustion engine vehicles to electric vehicles in the past 5 years. Looking back, all strategic decisions were interdependent and none of them was anticipated as a certainty or agreed upon by those affected or involved. The trajectory of electric mobility owed a lot to historical contingencies and strategic decision making in different action fields (industry, policy) (described in Kemp [72] and Dijk *et al.* [73]). It shows how beliefs and decisions of consumers, products and policy makers endogenously changed in socio-technical transitions.

10 The examples discussed are: intensive commercial forestry, administrative organization of cities, Soviet collectivization, colonial high-modernist agriculture in East Africa, and state villages in Ethiopia. They aimed at improving agriculture productivity or the human condition on the basis of (narrow bodies of) scientific knowledge and ‘a tendency to disallow other competing sources of judgment’ [77, p. 93].
